# Performance of Introducing Outdoor Cold Air for Cooling a Plant Production System with Artificial Light

**DOI:** 10.3389/fpls.2016.00270

**Published:** 2016-03-30

**Authors:** Jun Wang, Yuxin Tong, Qichang Yang, Min Xin

**Affiliations:** ^1^Institute of Environment and Sustainable Development in Agriculture, Chinese Academy of Agricultural ScienceBeijing, China; ^2^Key Laboratory of Energy Conservation and Waste Management of Agricultural Structures, Ministry of AgricultureBeijing, China

**Keywords:** coefficient of performance, energy saving, heat pump, lettuce, plant factory

## Abstract

The commercial use of a plant production system with artificial light (PPAL) is limited by its high initial construction and operation costs. The electric-energy consumed by heat pumps, applied mainly for cooling, accounts for 15–35% of the total electric-energy used in a PPAL. To reduce the electric-energy consumption, an air exchanger with low capacity (180 W) was used for cooling by introducing outdoor cold air. In this experiment, the indoor air temperature in two PPALs (floor area: 6.2 m^2^ each) was maintained at 25 and 20°C during photoperiod and dark period, respectively, for lettuce production. A null CO_2_ balance enrichment method was used in both PPALs. In one PPAL (PPAL_e_), an air exchanger (air flow rate: 250 m^3^·h^−1^) was used along with a heat pump (cooling capacity: 3.2 kW) to maintain the indoor air temperature at the set-point. The other PPAL (PPAL_c_) with only a heat pump (cooling capacity: 3.2 kW) was used for reference. Effects of introducing outdoor cold air on energy use efficiency, coefficient of performance (COP), electric-energy consumption for cooling and growth of lettuce were investigated. The results show that: when the air temperature difference between indoor and outdoor ranged from 20.2 to 30.0°C: (1) the average energy use efficiency of the air exchanger was 2.8 and 3.4 times greater than the COP of the heat pumps in the PPAL_e_ and PPAL_c_, respectively; (2) hourly electric-energy consumption for cooling in the PPAL_e_ reduced by 15.8–73.7% compared with that in the PPAL_c_; (3) daily supply of CO_2_ in the PPAL_e_ reduced from 0.15 to 0.04 kg compared with that in the PPAL_c_ with the outdoor air temperature ranging from −5.6 to 2.7°C; (4) no significant difference in lettuce growth was observed in both PPALs. The results indicate that using air exchanger to introduce outdoor cold air should be considered as an effective way to reduce electric-energy consumption for cooling with little effects on plant growth in a PPAL.

## Introduction

Recently, plant production systems with artificial light (PPAL) have been gradually used for plant growth in Asian countries, like China and Japan, due to their incomparable advantages of year round scheduled high-quality plant production, high resources use efficiencies, little environmental pollutions, etc. (Kozai, 2012; Fang, 2013). Unlike a greenhouse, the wall of a PPAL is constructed with opaque thermal insulated materials and artificial lighting is the sole light source for plant growth (Kozai et al., 2006; Li et al., 2012). Heat pumps are often used to remove extra heat generated from artificial lighting to maintain an optimum air temperature. Therefore, the operation cost of a PPAL is high and the commercial use of PPAL is limited. The cost of electric-energy consumed by artificial lighting and heat pumps, accounts for more than 30% of the total operation cost in a PPAL (Fang, 2013). The electric-energy consumed by heat pumps, accounts for 15–35% of the total electric-energy consumption in a PPAL, is mainly used for cooling (Nishimura et al., 2001; Ohyama et al., 2001). Therefore, reducing the electric-energy consumption of heat pumps would be helpful for reducing the operation cost of a PPAL.

Several methods were investigated to reduce the electric-energy consumed by heat pumps for cooling: (1) employing light devices with low heat generation, such as light emitting diode (e.g., Poulet et al., 2014), for reducing heat load in a PPAL, (2) changing light intensity at different plant growth stages (e.g., Tong and Yang, 2014), (3) employing movable light systems (e.g., Li et al., 2014), and (4) introducing seasonal ice storage system for cooling (Yang et al., 2012), etc. However, few studies have been reported on reducing electric-energy consumption for cooling by improving the coefficient of performance (COP) or shortening the operation time of heat pumps working with low COP. It has been reported that high COP for cooling is usually achieved when cooling load is 60–80% of the cooling capacity of a heat pump (Tong et al., 2013). Too low/high heat load reduces the COP. Low heat load also causes frequent ON/OFF operation of heat pumps, which reduces the COP. Outdoor air, whenever its temperature is below the air temperature in the PPAL, is a potential cold source for cooling the air temperature in a PPAL. And therefore direct air exchange may be more energy-efficient than using a heat pump. The main drawback of direct air exchange is that CO_2_ utilization efficiency would decrease when indoor CO_2_ concentration needs to be kept at high levels (e.g., 1000 μmol mol^−1^) because some of the injected CO_2_ would be lost during the air exchange process (Tingey et al., 2000). Without CO_2_ enrichment, CO_2_ depletion (50–200 μmol mol^−1^) could occur during photoperiod, even with natural ventilation (Sanchez-Guerrero et al., 2005). Under such conditions, CO_2_ concentration may become a limiting environmental factor for photosynthesis (Thongbai et al., 2010). Therefore, a null CO_2_ balance enrichment method, where CO_2_ concentration is maintained at the same as outdoor level, is proposed to keep high utilization efficiency of supplied CO_2_ when the outdoor cold air is introduced into a PPAL (Kozai, 2009).

In this study, we investigated air exchanging aided by a heat pump as a primary cooling method. The performance of the cooling method was compared with the cooling method that depended solely on a heat pump. To avoid CO_2_ depletion and keep a high CO_2_ utilization efficiency, the null CO_2_ balance enrichment method was used. Two PPALs employed each of the two cooling methods. The objectives of this study are: (1) to analyze the feasibility of saving electric-energy by exchanging air with outdoor cool air in comparison with using only the heat pump; (2) to examine whether air exchanging would impact the amount of supplied CO_2_ if null CO_2_ balance enrichment method is used; and (3) to determine whether introducing outdoor air would have any negative effects on plant growth.

## Materials and methods

### Plant production system with artificial light (PPAL)

**Figure 1:**
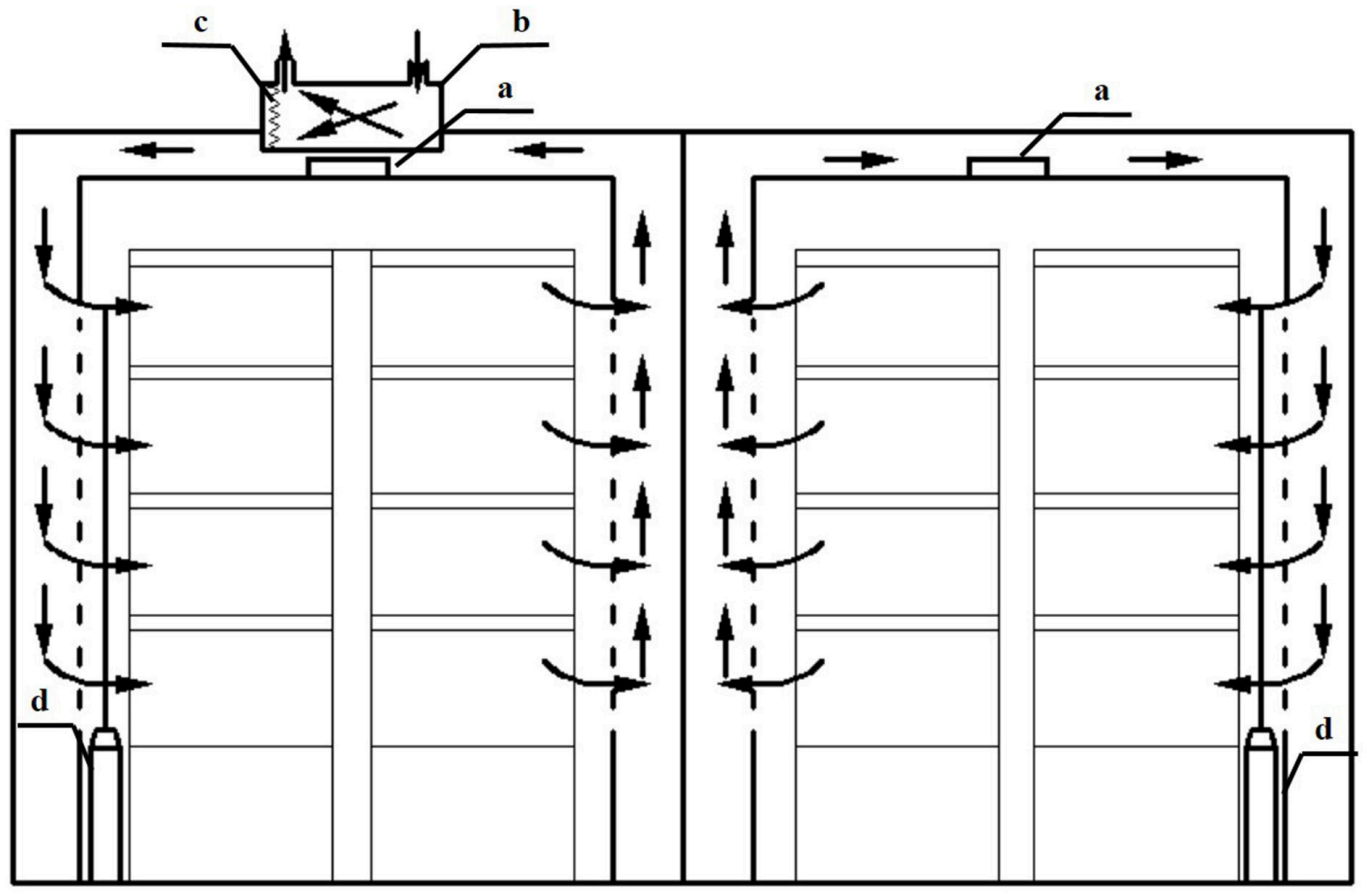
**Schematic diagram of the plant factories used in the experiment**. a: heat pump installed inside the PPAL; b: air exchanger installed inside the ceiling of the PPAL; c: air filter; d: CO_2_ gas cylinder.

Two PPALs were used in this experiment. Each PPAL with a volume of 11.7 m^3^ (2.2 m wide, 2.8 m long and 1.9 m high) was built in 2012 and located in China Academy of Agricultural Sciences, Beijing. The Plan view of the PPALs is shown in Figure 1. Each PPAL was installed two plant cultivation modules with three layers. The dimension of the plant cultivation modules was 2.1 × 0.7 × 1.8 m. Each layer was equipped with a cultivation bed (2.0 m long, 0.6 m wide, and 0.06 m deep) and fluorescent lamps (24 W, NVC-YZ24-T5, Guangdong, China), 0.4 m above the cultivation bed. In the culture beds, nutrient solution was circulated continuously by water pumps. Styrofoam boards were floated on the nutrient solution to support lettuce plants. Each PPAL was equipped with an air source heat pump for cooling (cooling capacity: 3.2 kW, FTXN32KV2C, Daikin, Industries. Ltd, Japan) and a CO_2_ gas cylinder for CO_2_ enrichment. On the ceiling of the experimental PPAL (PPAL_e_), one air exchanger (capacity: 180 W, air flow rate: 250 m^3^·h^−1^, FY-25LD2C, Guangdong, China) and an air filter (FY-BFG062C, Panasonic Ecology Systems Guangdong Co., Ltd) was installed for exchanging indoor warm and humid air with outdoor cold and dry air. Another PPAL (PPAL_c_) with only the heat pump was used as reference.

### Plant materials and growth conditions

Lettuce plants (*Lactuca* sativa var.) were grown in the PPALs during the experiment. Lettuce seeds were sown in a plastic tray under LED light (80 μmol·m^−2^·s^−1^) and irrigated with tap water once a day. Fifteen days after sowing, uniform seedlings were transplanted into the styrofoam boards with a density of 25 plants·m^−2^ and grown hydroponically using modified Hoagland's solution, whose chemical composition was as follows (mg·L^−1^): Ca(NO_3_)_2_·4H_2_O, 945; KNO_3_, 607; NH_4_H_2_PO_4_, 115; MgSO_4_·7H_2_O, 495; Na_2_Fe-EDTA, 30; MnSO_4_·H_2_O, 1.61; CuSO_4_·5H_2_O, 0.08; ZnSO_4_·7H_2_O, 0.22; (NH4)_6_Mo_7_O_24_·4H_2_O, 0.02; H_2_BO_3_, 2.86. The nutrient solution was renewed once a week and the electrical conductivity of the solution was adjusted to 2.0 dS·m^−1^. Nutrient solution pH was maintained at 6.0 to 6.5. During the experiment, indoor air temperature was set at 25 and 20°C during photoperiod and dark period, respectively. Photosynthetic photon flux (*PPF*) measured at a distance of 10 cm up from cultivation beds was 150 μmol·m^−2^·s^−1^ with a photoperiod of 12 h·d^−1^ (dark period: 21:00–09:00). Indoor CO_2_ concentration was always kept at the same as outdoor level.

**Table:** 


Notation	
*Symbols*	
Variables	
*COP*	Coefficient of performance for heating
*C*	Daily use of CO_2_, kg
*EUE*	Energy use efficiency
*E*	Electricity consumption rate of air exchanger, W
*i*	Enthalpy, kJ kg^−1^
*P*	Electricity consumption rate of heat pump, W
*Q*	Heat energy, W
*q*	Air flow rate, m^3^·s^−1^
*R*	Reduction in daily use of CO_2_
*SHF*	Sensible heat factor
*T*	Air temperature, °C
Constants	
ρ	Specific weight of air density of dry air, kg·m^−3^
λ	Latent heat of water vaporization, J·Kg^−1^
Subscripts	
*ax*	Air exchanger
*c*	In the PPAL_c_
*d*	At the air discharge
*e*	In the PPAL_e_
*h*	heat energy
*hp*	Heat pump
*l-hp*	Latent heat extracted by heat pump
*l-ax*	Latent heat extracted by air exchanger
*i*	Indoor air
*o*	Outdoor air
*s*	At the air suction

### Experimental setup

In the PPAL_e_, indoor air temperature (*T*_*i*_) was allowed to maintain at around the set point of 25 and 20°C during photoperiod and dark period, respectively, within the range from *T*_*l*_ to *T*_*h*_, where the *T*_*l*_ was the low air temperature set point and *T*_*h*_ was the high temperature set point. *T*_*l*_ was 23 and 18°C during photoperiod and dark period, respectively; *T*_*h*_ was 27 and 22°C during photoperiod and dark period, respectively. The air temperature dead band was configured to avoid frequent ON/OFF operation of the air exchanger and heat pump for cooling. *T*_*i*_ in the PPAL_e_ was controlled by the air exchanger aided by the heat pump. When the outdoor air temperature (*T*_*o*_) was lower than *T*_*l*_, and *T*_*i*_ was higher than the temperature set point, the outdoor cold and dry air was introduced by the air exchanger for cooling. If *T*_*i*_ went above *T*_*h*_, the air exchanger was turned off, and the heat pump started to operate for cooling until *T*_*i*_ decreased to the set temperature. If *T*_*i*_ dropped below *T*_*l*_, the air exchanger was also automatically turned off. If *T*_*o*_ was higher than *T*_*h*_, the heat pump was operated for cooling. In the PPAL_c_, only the heat pump was used to maintain the indoor air temperature at the set point of 25 and 20°C during photoperiod and dark period, respectively.

During photoperiod, a null CO_2_ balance enrichment method was employed in both PPALs. The CO_2_ supply system consisted of a CO_2_ gas cylinder, an electronic mass flow meter (CMS 0020/0050), CO_2_ distribution perforated plastic tubes (3 mm, polyethylene) network. The CO_2_ distribution plastic tubes reached out to each layer to distribute CO_2_ uniformly. In the PPAL_e_, CO_2_ was supplemented by outdoor air when the air exchanger was operating. But whenever the indoor CO_2_ concentration was 50 μmol·mol^−1^ lower than outdoor due to reduced operation of air exchanger, the CO_2_ from the gas cylinder was released into the PPAL_e_. While in the PPAL_c_, only the CO_2_ from the gas cylinder was used to keep the indoor CO_2_ concentration at the same as outdoor level. The experiment was conducted from November 13th to December 18th in 2014.

### Calculations

#### Coefficient of performance (COP) for cooling of the heat pump

COP of the heat pump for cooling a PPAL was defined as:

(1) $$COP=\frac{Q_{hp}}{P}$$

where *Q*_*hp*_: heat energy removed rate by the heat pump (W), *P*: electric-energy consumption rate of the heat pump (W).

Heat energy removed rate by the heat pump was calculated by the general known equation:

(2) $$Q_{hp}=q\cdot \rho \cdot (i_{s}-i_{d})$$

where *q*: air flow rate of the heat pump (m^3^·s^−1^); ρ: density of dry air (kg (D.A.)·m^−3^); *i*_*s*_: enthalpy at the air suction of the internal unit of the heat pump (J·kg^−1^ (D.A.)); *i*_*d*_: enthalpy at the discharge ports of the internal unit of the heat pump (J·kg^−1^ (D.A.)).

The sensible heat factor of the heat pump (*SHF*_*hp*_) was defined as:

(3) $$SHF_{hp}=1-\frac{Q_{l-hp}}{Q_{hp}}$$

where

(4) $$Q_{l-hp}=\lambda \cdot q\cdot \rho \cdot (x_{s}-x_{d})$$

is the latent heat load (W), with λ: latent heat of water vaporization (2.5 × 10^6^ J·kg^−1^ at 20°C; ASHRAE, 2001); *x*_*s*_, *x*_*d*_: absolute humidity at the air suction and discharge ports of the internal unit of the heat pump (kg·kg^−1^ (D.A.)).

#### Electric-energy use efficiency of the air exchanger

The electric-energy use efficiency of air exchanger (EUE) was defined as:

(5) $$EUE=\frac{Q_{ax}}{E}$$

where *Q*_*ax*_: heat energy removed rate by the air exchanger (W), *E*: electricity consumption rate of the air exchanger (W).

Heat energy removed rate by the air exchanger was calculated similar to Equation (2):

(6) $$Q_{ax}=q\cdot \rho \cdot (i_{i}-i_{o})$$

where *i*_*i*_: enthalpy of the indoor air (J·kg^−1^ (D.A.)); *i*_*o*_: enthalpy of the outdoor air(J·kg^−1^ (D.A.)).

The sensible heat factor of air exchanger (*SHF*_*ax*_) can be estimated by:

(7) $$SHF_{ax}=1-\frac{Q_{l-ax}}{Q_{ax}}$$

Where

(8) $$Q_{l-ax}=\lambda \cdot q\cdot \rho \cdot (x_{i}-x_{o})$$

is the latent heat load (W); *x*_*i*_: absolute humidity of the air expelled from the PPAL_e_ (kg·kg^−1^ (D.A.)); *x*_*o*_: absolute humidity of the outdoor air entering the PPAL_e_ (kg·kg^−1^ (D.A.)).

#### Reduction in the amount of supplied CO_2_

The reduction in the amount of supplied CO_2_(R) was calculated by:

(9) $$R=C_{c}-C_{e}$$

Where *C*_*c*_: daily amount of CO_2_ supplemented to the PPALc (kg); and *C*_*e*_: daily amount of CO_2_ supplemented to the PPAL_e_ (kg).

### Measurements

#### Environmental conditions inside and outside the PPALs

The inside air temperature and relative humidity of both the PPAL_e_ and PPAL_c_ were measured by sensors (TR-72WF, T&D, Co. Japan; precision: air temperature ±0.3°C, relative humidity ±3%). Three sensors were set at a height of 1.5 m from the ground at three measuring points in the middle of each PPAL. The air temperature and relative humidity at the air suction and air discharge ports of the internal unit of each heat pump were measured using sensors (TR-72WF, T&D, Co. Japan; precision: air temperature ±0.3°C, relative humidity ±3%). Vapor pressure deficit (VPD) was determined from the measurements of the air temperature and relative humidity, as the method described in Prenger and Ling (2001). The inside CO_2_ concentration was measured by an infrared type CO_2_ analyzer (GMT 222, Vaisala Oyj, Helsinki, Finland). The CO_2_ analyzer was set at 1.5 m above floor level in the middle of each PPAL. All the above data were automatically recorded every minute with a wireless data collection system. The air flow rate at the air discharge port of the internal unit of each heat pump was measured manually once every second using an air flow meter (Model 6533, Kanomax, Japan).

The outdoor air temperature, relative humidity (TR-72WF, T&D, Co. Japan; precision: air temperature ±0.3°C, relative humidity ±3 %) and CO_2_ concentration(GMT 222, Vaisala Oyj, Helsinki, Finland) were recorded every minute in a small weather station 5 m away from the experimental PPAL. All the data were recorded every minute.

#### Energy consumption in both PPALs

The electric-energy consumption rates of heat pumps and air exchanger were measured by wattmeters (KWm8115, Panasonic Electric Works, Japan) and recorded every minute.

#### Daily use of CO_2_ supplemented to both PPALs

Daily use of CO_2_ supplemented to both PPALs were determined from continuously measurement of the weight of the CO_2_ cylinders, using electronic scales (FZ-TCS50, Julin, Instruments, Co., Xiamen, China).

#### Measurement of lettuce plant growth and chlorophyll concentration

At the 25 and 35 days after transplanting, lettuce plants were randomly sampled to measure their growth. Fifteen plants from each PPAL were harvested each time to measure their fresh weights. Dry weights were measured after being dried at 80°C for 72 h.

Samples were excised from the leaves of 10 plants at a similar position for each treatment. Leaves were weighed out in 0.1–0.2 g (fresh weight). The extractions were performed using 10 ml (V) of 80% acetone until the leaf turned white. The optical density was measured with UV-1800 spectrophotometer (Shimadzu, Japan) at 663 nm (OD663) and at 645 nm (OD645) for chlorophyll a (Chl a) and chlorophyll b (Chl b). The chlorophyll concentrations (Chl) were determined as the method described in Lichtenthaler and Wellburn (1983).

#### Statistical analysis

Statistical differences among the treatments were analyzed by the least significant difference (LSD) test (*p* < 0.05) when analysis of variance (ANOVA) by SPSS software (SPSS for Windows, SPSS Inc., USA) indicated treatment significance.

## Results

### Energy use efficiencies of air exchanger and heat pumps

**Figure 2:**
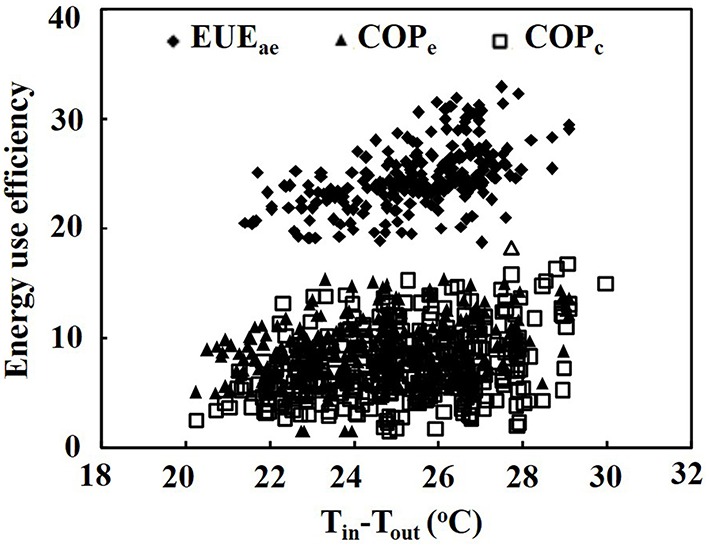
**Energy use efficiency of the air exchanger and heat pumps as affected by the air temperature difference between indoor and outdoor**. EUE, energy use efficiency of the air exchanger; COP_e_, energy use efficiency of the heat pump in the PPAL_e_; COP_c_, energy use efficiency of the heat pump in the PPAL_c_.

Both the EUE of air exchanger and the COP of heat pumps for cooling increased with the increase in air temperature difference between indoor and outdoor. When the air temperature difference between indoor and outdoor ranged from 20.2 to 30.0°C, the EUE of the air exchanger for cooling the PPAL_e_ ranged from18.6 to 32.9 with an average of 25.6, the COP of the heat pump in the PPAL_e_ ranged from 4.2 to 18.2 with an average of 9.0, and the COP of the heat pump in the PPAL_c_ ranged from 1.6 to 16.8 with an average of 7.5 (Figure 2). The average EUE of air exchanger was 2.8 and 3.4 times greater than the average COP of the heat pumps in the PPAL_e_ and PPAL_c_, respectively.

### SHF of air exchanger and heat pumps

**Figure 3:**
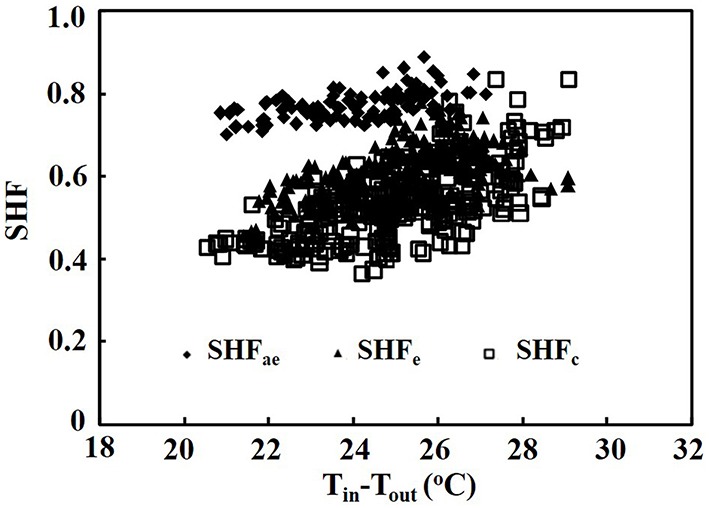
**Sensible heat factor (SHF) of the air exchanger and heat pumps as affected by the air temperature difference between indoor and outdoor**. SHF_ae_, sensible heat factor of the air exchanger; SHF_e_, sensible heat factor of the heat pump in the PPAL_e_; SHF_c_, sensible heat factor of the heat pump in the PPAL_c_.

When the air temperature difference between indoor and outdoor ranged from 20.2 to 30.0°C, the SHF of the air exchanger ranged from 0.7 to 0.9, the SHF of the heat pump in the PPAL_e_ ranged from 0.5 to 0.7, while the SHF of the heat pump in the PPAL_c_ ranged from 0.4 to 0.8 (Figure 3).

### Electric-energy consumption of air exchanger and heat pumps

**Figure 4:**
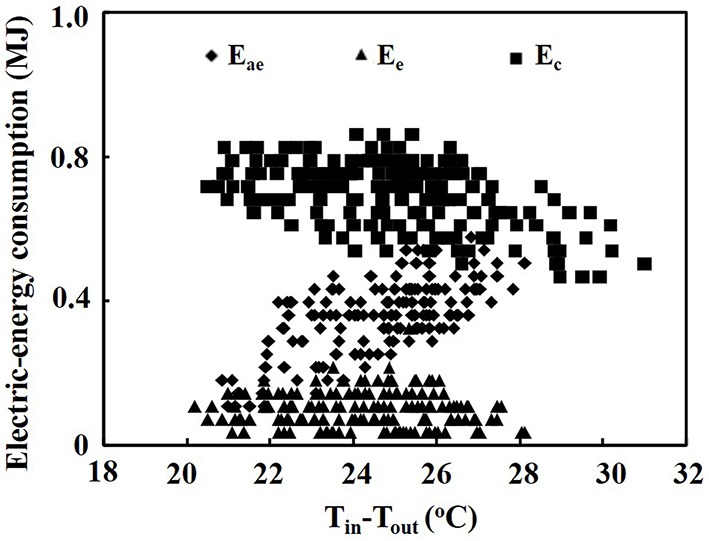
**Electric-energy consumption of the air exchanger and heat pumps as affected by the air temperature difference between indoor and outdoor**. E_ae_, electric-energy consumption of the air exchanger; E_e_, electric-energy consumption of the heat pump in the PPAL_e_; E_c_, electric-energy consumption of the heat pump in the PPAL_c_.

When the air temperature difference between indoor and outdoor ranged from 20.2 to 35.7°C, the hourly electric-energy consumption of the air exchanger ranged from 0.11 MJ to 0.58 MJ, while that of the heat pump in the PPAL_e_ ranged from 0.32 to 0.04 MJ, and that of the heat pump in the PPAL_c_ ranged from 0.86 to 0.47 MJ (Figure 4). The hourly electric-energy consumption in the PPAL_e_ was 15.8–73.7% lower than that in the PPAL_c_.

### Reduction in the use of supplemental CO_2_

**Figure 5:**
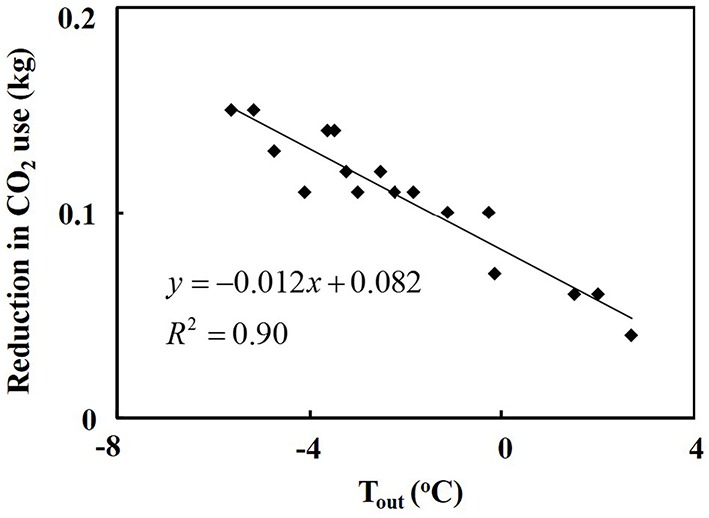
**Reduction of daily CO_2_ supplied in the PPAL_*e*_ compared with that in the PPAL_*c*_ as affected by outdoor air temperature**.

The PPAL_e_ required 0.04–0.15 kg less CO_2_ per day to replenish the CO_2_ depletion than the PPAL_c_, when the daily average outdoor air temperature ranged from −5.6 to 2.7°C (Figure 5).

### Environmental conditions in both PPALs

**Figure 6:**
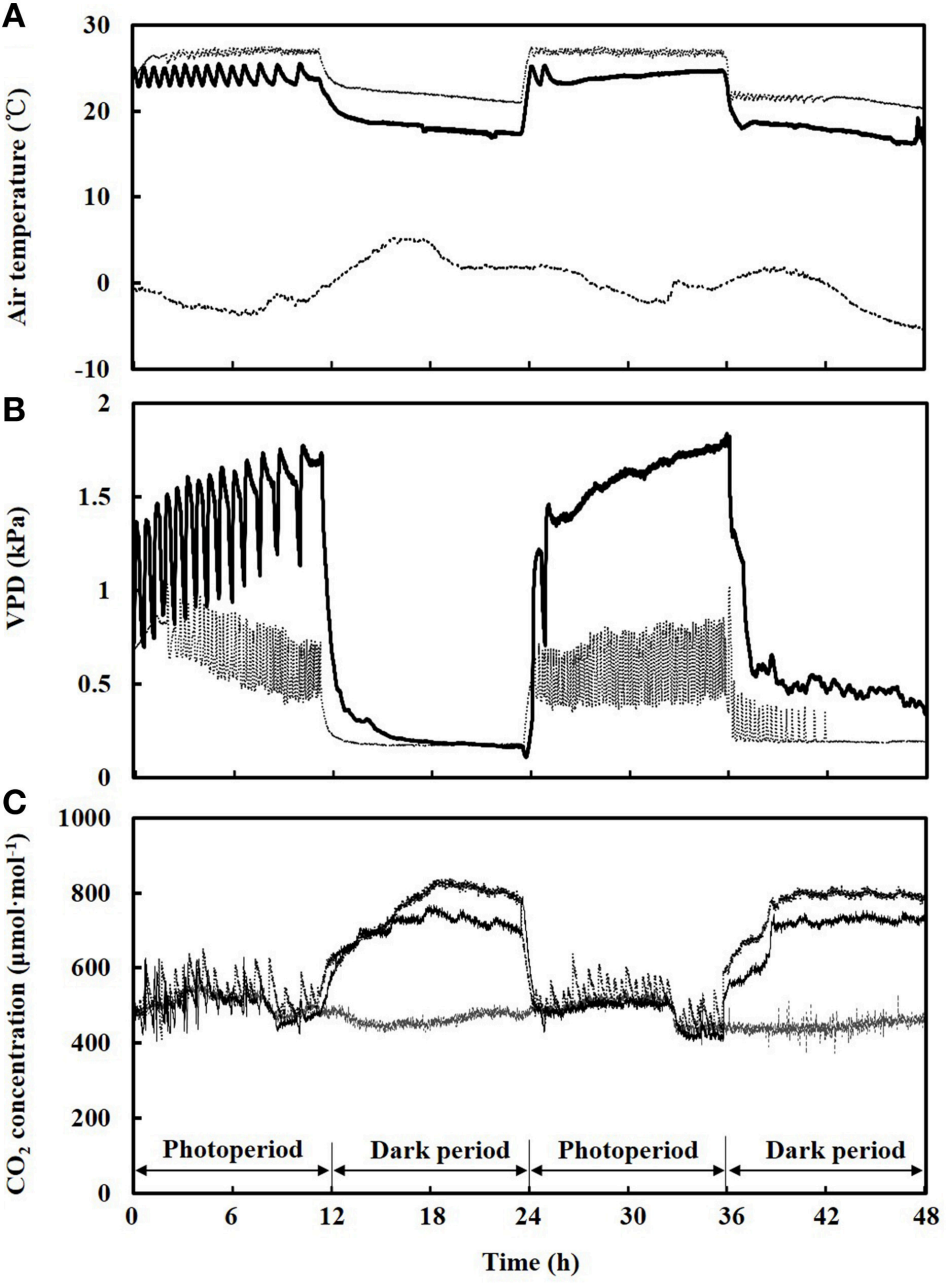
**Time course of air temperature (A), vapor pressure deficit (VPD) (B) and CO_2_ concentration (C)**. ___: environmental conditions in the PPAL_e_; ……: environmental conditions in the PPAL_c_; - - -: outdoor environmental conditions.

The air temperature inside both PPALs could be controlled within the acceptable ranges (23 to 27°C during photoperiod and 18 to 22°C during dark period, respectively). The air temperature inside the PPAL_e_ was lower than that of PPAL_c_ with a highest different value of 4.7°C. The high fluctuation of air temperature inside the PPAL_e_ was observed especially during lower outdoor air temperature (Figure 6A).

The VPD in the PPAL_e_ ranged from 0.5 to 1.8 kPa during photoperiod and from 0.2 to 1.0 kPa during dark period. While the VPD in the PPAL_c_ ranged from 0.2 to 1.8 kPa during photoperiod and from 0.2 to 1.0 kPa during dark period (Figure 6B).

CO_2_ concentration during photoperiod in both PPALs could be maintained at around outdoor level in this experiment. Average CO_2_ concentration during photoperiod was 493 and 510 μmol mol^−1^ in the PPAL_e_ and PPAL_c_, respectively, while the average outdoor CO_2_ concentration was 498 μmol mol^−1^ (Figure 6C). During dark period, CO_2_ concentration in the PPAL_e_ increased from 472 to 769 μmol·mol^−1^, while that in the PPAL_c_ increased from 522 to 841 μmol·mol^−1^. CO_2_ concentration during dark period was lower in the PPAL_e_ than that in the PPAL_c_ during this experiment.

### Lettuce growth in both PPALs

**Figure 7:**
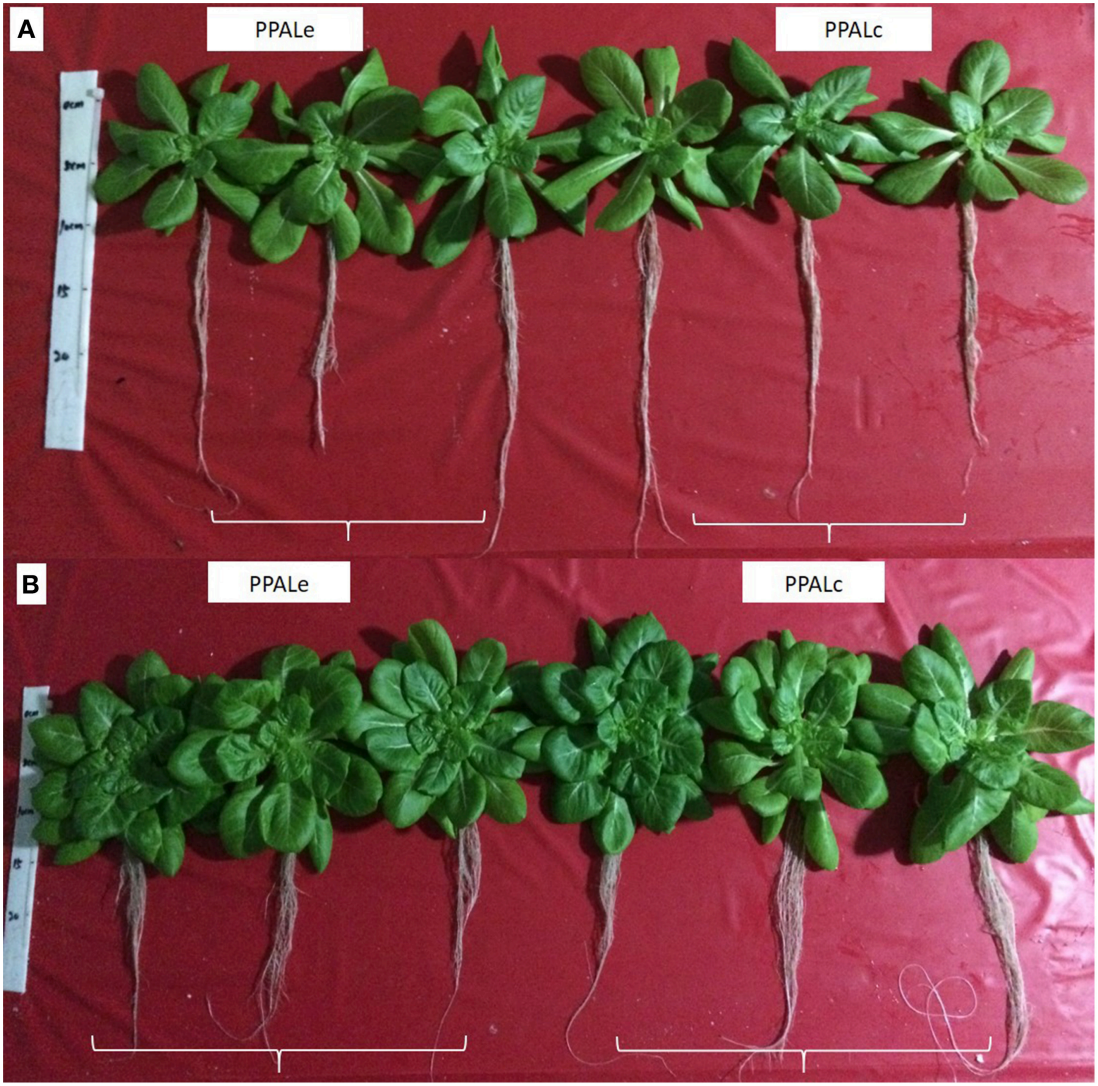
**Growth images of lettuce on DAT15 (A) and DAT30 (B) in the PPAL_*e*_ and PPAL_*c*_**.

**Figure 8:**
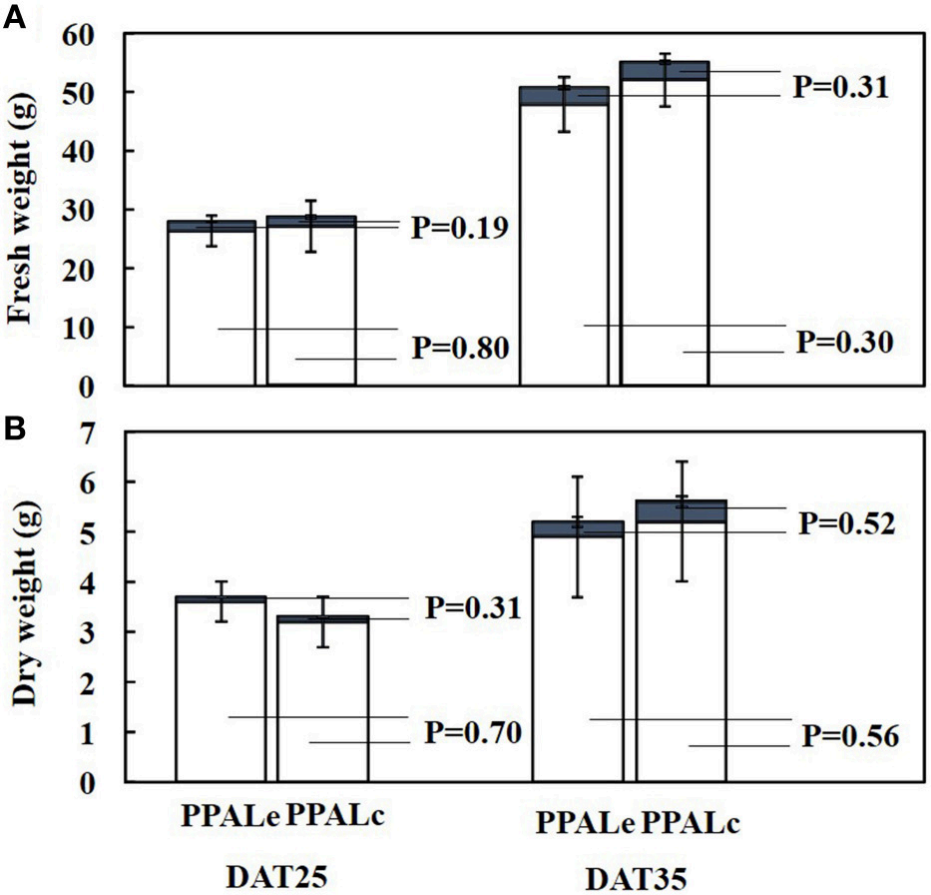
**Fresh (A) and dry weights (B) of lettuce on DAT25 and DAT35 in PPAL_*e*_ and PPAL_*c*_**. Open and filled bars respect shoot weight and root weight, respectively. Values were the means of six replicates with standard errors shown by vertical bars. Statistical differences among the treatments were analyzed by LSD test (*p* < 0.05).

**Figure 9:**
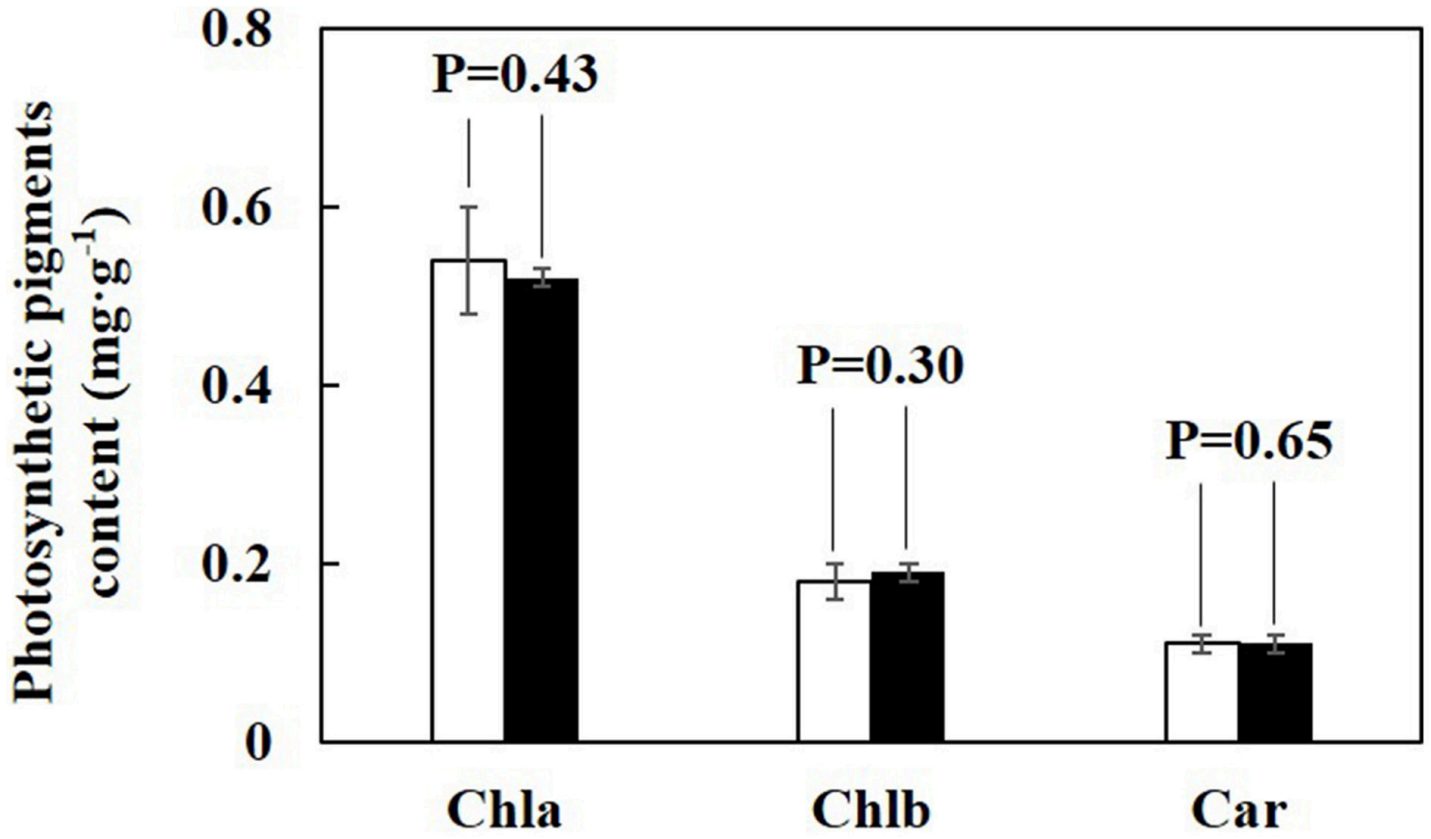
**Photosynthetic pigments (chlorophyll and carotenoid) contents of harvested lettuce on DAT35 in both PPALs**. Open and filled bars respect photosynthetic pigments content of lettuce in the PPAL_e_ and PPAL_c_, respectively. Values were the means of six replicates with standard errors shown by vertical bars. Statistical differences among the treatments were analyzed by LSD test (*p* < 0.05).

The growth images of lettuce on DAT15 and DAT30 in PPALe and PPALc were showed in Figures 7A,B, respectively. Figure 8 presents the fresh and dry weights measured at 25 and 35 days after transplanting. No significant differences of fresh or dry weight of lettuce plants were found between the two PPALs. By calculating from data used in Figure 8, the ratio of shoot fresh weight to dry weight in PPAL_e_ and PPAL_c_ were 16.4 and 16.3 g·g^−1^, respectively, while the ratio of root fresh weight to dry weight in PPAL_e_ and PPAL_c_ were 29.1 and 32.3 g·g^−1^, respectively. There were no significant differences between the two PPALs. Photosynthetic pigments of lettuce plants in both PPALs also showed no significant difference (Figure 9).

## Discussion

### Feasibility of saving electric-energy by air exchanging with outdoor air for cooling

In the present experiment, the high EUE of air exchanger indicates that air exchanger with a low capacity is more energy-efficient for cooling in a PPAL than the heat pump with a high capacity under the experimental conditions. In the present experiment, both the EUE of air exchanger and the COP of heat pumps for cooling increased with decreasing outdoor air temperature when the indoor air temperature could be maintained at around the set point, which agrees with previous reports (e.g., Tong et al., 2013). The reason of higher EUE of the air exchanger in comparison with the COP of the heat pumps probably was that the outdoor air with low temperature and humidity was introduced into indoor directly by using air exchanger with low capacity. The COP of the heat pump in the PPAL_e_ was slightly higher than the COP of heat pump in the PPAL_c_, probably because the operation time of the heat pump in the PPAL_e_ in low heat load was reduced by introducing outdoor cold air.

In Figure 2, the scattered data for EUE of the air exchanger and COP of both heat pumps were probably because the EUE and COP were not only affected by the air temperature but also affected by the relative humidity of indoor and outdoor air (Tong et al., 2010). Thus, the air exchanger and heat pump not only extracted sensible heat but also latent heat from the PPALs. The SHF value represents the ratio of the sensible heat energy to the total heat energy removed from the PPALs by air exchanger and heat pumps. The higher SHF value of the air exchanger indicated that mainly sensible heat contributed to the total heat energy extracted from the PPAL_e_, because introducing outdoor dry air reduced indoor-outdoor relative humidity difference. The SHF of the heat pump in the PPAL_c_ was slightly lower than that in the PPAL_e_ (Figure 3), probably because the higher indoor relative humidity increased the ratio of latent heat energy to the total heat energy extracted from the PPAL_c_.

### Saving electric-energy by using the air exchanger

Hourly electric-energy consumption of the air exchanger and heat pumps as affected by air temperature difference between indoor and outdoor is shown in Figure 4. The electric-energy consumption of the heat pump both in the PPAL_c_ and the PPAL_e_ decreased with increasing air temperature difference between indoor and outdoor due to the increased COP. The hourly electric-energy consumption of the heat pump in the PPAL_e_ was much lower than that of the heat pump in the PPAL_c_. Compared with the heat pump in the PPAL_c_, saving-energy of the heat pump in the PPAL_e_ became more pronounced with increasing air temperature difference between indoor and outdoor. The above results indicated that the operation time of the heat pump in the PPAL_e_ was significantly shortened, particularly under large air temperature difference. The hourly electric-energy consumption of air exchanger increased with increasing air temperature difference between indoor and outdoor although the EUE increased. This was because with decreasing outdoor air temperature, operation time of the air exchanger increased since the indoor air temperature could be controlled at the set point using only air exchanger for cooling.

### Reduction in the use of supplemental CO_2_

In the experiment, to avoid high CO_2_ depletion and low CO_2_ utilization efficiency, CO_2_ concentration was replenished to keep the concentration at outside level. The reduction in daily supply of CO_2_ in the PPAL_e_ was because CO_2_ gas from outdoor air was supplied to the indoor air when the air exchanger was used and the pure CO_2_ was used whenever the indoor CO_2_ concentration could not be kept at outside level, while in the PPAL_c_, only pure CO_2_ was used to keep the indoor CO_2_ concentration at outside level. Increasing operation time of the air exchanger for introducing outdoor cold air could save CO_2_ supplied into a PPAL with the null CO_2_ balance enrichment method. With increasing outdoor air temperature the operation time of the air exchanger decreased as shown in Figure 4, so then more pure CO_2_ was needed.

### Environmental conditions in both PPALs

Time course of air temperatures inside both PPALs shown in Figure 6A indicates that the air exchanger could be used for cooling in most of the time since the heat pump was operated for cooling when the indoor air temperature could not be controlled below 27 and 22°C during photoperiod and dark period, respectively. The fluctuation of air temperature inside the PPAL_e_ was because different control strategies of the air exchanger and heat pumps were employed in this experiment. Heat pumps employed a proportional-integral-derivative (PID) control method, while the air exchanger employed a simple ON/OFF control method.

CO_2_ concentration during photoperiod in both PPALs could be maintained at around outdoor level in this experiment, with the reasonable fluctuations of CO_2_ concentration in both PPALs caused by ON/OFF operation of the CO_2_ supplemental devices. To reduce the fluctuations in CO_2_ concentration, PID control method should be employed instead of ON/OFF control method. Compared to CO_2_ concentration in the PPAL_c_, lower CO_2_ concentration in the PPAL_e_ during dark period was caused by the amount of CO_2_ leakage increasing with increasing air exchange rate due to the operation of the air exchanger.

### Lettuce growth in both PPALs

No significant differences of fresh/dry weights and photosynthetic pigments of lettuce plants were found despite of the differences in environmental conditions of the two PPALs. The air temperatures inside both PPALs were controlled within the optimum ranges recommended (Li et al., 2012; Chen et al., 2014; Tong and Yang, 2014), but the air temperature inside the PPAL_c_ was slightly higher than that inside the PPAL_e_. High air temperature during photoperiod significantly improved plant growth, while high air temperature during dark period had adverse effects on plant growth because it would enhance dark respiration (Peet and Bartholomew, 1996; Morales et al., 2003).

A similar VPD ranged from 0.2 to 1.0 kPa was observed in both PPALs during dark period. Grange and Hand (1987) quoted that a VPD in the range of 0.2–1.0 kPa has little effect on the physiology and development of crops, while during photoperiod, the VPD in the PPAL_e_ was much higher than that in the PPAL_c_, because the very dry outdoor air was introduced into the PPAL_e_ when the air exchanger was used. According to Hoffman (1979), an increase in VPD from 1 to 1.8 kPa determines the major reduction in plant growth on several crops, and this could be probably due to the depression of photosynthesis (Xu et al., 1991), related to the reduction of stomatal conductance (Grange and Hand, 1987). In contrast, some studies reported that low VPD positively affects dry matter accumulation and can also promote the incidence of calcium related physiological disorders in leaves (Janse and Welles, 1984; Holder and Cockshull, 1990; Kreij, 1996; Dorais et al., 2004).

The average CO_2_ concentration in both PPALs was maintained at approximately the same as outdoor level during photoperiod, although CO_2_ concentration in the PPAL_c_ was slightly higher than that in the PPAL_e_ during dark period. Based on above discussion, the lettuce plants growth in this experiment might be affected by different environmental conditions, but positive effects because of higher air temperature during photoperiod were all offset by negative effects of higher air temperature during dark period and lower VPD in the PPAL_c_.

### Economic benefit analysis of introducing outdoor cold air for cooling together with null CO_2_ balance enrichment method

Costs for electric-energy consumed by the air exchanger and heat pumps were analyzed based on an industrial electricity cost of 0.81 RMB·kWh^−1^ and data in Figure 4. Electric-energy cost for cooling in the PPAL_e_ ranged from 0.10 to 1.89 RMB·h^−1^, with an average of 1.14 RMB·h^−1^, while the electric-energy cost for cooling in the PPAL_c_ ranged from 1.36 to 2.52 RMB·h^−1^, with an average of 2.04 RMB·h^−1^, when the air temperature difference between indoor and outdoor ranged from 20.2 to 35.7°C. The above results indicated that about 0.9 RMB·h^−1^ on average could be saved in the PPAL_e_ than that in the PPAL_c_ under the experimental conditions.

Cost for reduction in CO_2_ supplied in both PPALs was analyzed based on CO_2_ (purity of 99.9%) cost of 0.71 RMB·kg^−1^ and data in Figure 5. Reduction in CO_2_ cost ranged from 0.03 to 0.11 RMB·d^−1^, with an average of 0.08 RMB·d^−1^, when the daily average outdoor air temperature ranged from −5.6 to 2.7°C.

As discussed in sections of “Saving electric-energy by using the air exchanger” and “Reduction in the use of supplemental CO_2_,” the economic benefit of introducing outdoor cold air for cooling together with null CO_2_ balance enrichment method was significantly affected by the indoor and outdoor environmental conditions. Greater economic benefit can be achieved with increasing operation time of the air exchanger. Furthermore, the present experiment was conducted in a model PPAL with a small volume of 11.7 m^3^, while the volume of a commercial used PPAL is usually larger than 1000 m^3^. Thus, in a commercial used PPAL with a volume of 1000 m^3^, the saved electric energy and CO_2_ cost can be 76.92 RMB·h^−1^ and 6.84 RMB·d^−1^, respectively. However, the actual economic benefit in a commercial PPAL should be further confirmed in future researches.

## Conclusion

To reduce the electric-energy consumption for cooling in a PPAL, an air exchanger was employed aided by a heat pump for cooling by introducing outdoor air whenever the indoor air temperature was lower than outdoor air temperature. To avoid a high depletion of CO_2_ concentration and keep a high supplied CO_2_ utilization efficiency, a null CO_2_ balance enrichment method was used in this experiment. Experimental results showed that when the air temperature difference between indoor and outdoor ranged from 20.2 to 30.0°C: (1) the average EUE of the air exchanger was 2.8 and 3.4 times greater than the COP of the heat pumps in the PPAL_e_ and PPAL_c_, respectively; (2) the hourly electric-energy consumption in the PPAL_e_ was reduced by 15.8–73.7% compared with that in the PPAL_c_; (3) the daily use of supplemental CO_2_ in the PPAL_e_ was greatly reduced from 0.15 to 0.04 kg compared with that in the PPAL_c_. Operating the air exchanger did not affect the growth of lettuce. Overall, introducing cold air by air exchanger proved to be an effective cooling method for PPALs, however, auxiliary cooling devices are needed (such as heat pump) whenever air exchanger could not meet the cooling demand.

## Author contributions

JW carried out the measurements, data analysis and drafted the manuscript. MX participated in part of measurements and data analysis. YT and QY made substantial guide about experiment design, and critically revised the manuscript.

### Conflict of interest statement

The authors declare that the research was conducted in the absence of any commercial or financial relationships that could be construed as a potential conflict of interest.
